# Critical Global Health: Responding to Poverty, Inequality and Climate Change: Comment on "Politics, Power, Poverty and Global Health: Systems and Frames"

**DOI:** 10.15171/ijhpm.2016.157

**Published:** 2017-01-03

**Authors:** David McCoy

**Affiliations:** Centre for Primary Care and Public Health, Queen Mary University, London, UK.

**Keywords:** Politics, Power, Poverty, Global Health

## Abstract

A recent article by Sol Benatar calls on the global health community to reassess its approach to twin crises of global poverty and climate change. I build on his article by challenging mainstream narratives that claim satisfactory progress in efforts to reduce poverty and improve health for all, and arguing that any eradication of poverty that is consistent with environmental sustainability will require a more explicit emphasis on the redistribution of power and wealth. I suggest that the global health community has been largely socialised into accepting that progress and future solutions can be attained through more neoliberal development, technological advancement and philanthropic endeavour and that a more critical global health is required. I propose three steps that the global health community should take: first, create more space for the social, political and political sciences within global health; second, be more prepared to act politically and challenge power; and third, do more to bridge the global-local divide in recognition of the fact that progressive change requires mobilisation from the bottom-up in conjunction with top-down policy and legislative change.

Sol Benatar’s recent article^1^ in this journal raises a number of observations about the politics of global health and how priorities and needs are framed. Here I expand upon two points from his article, and raise questions for the community of academics, policy-makers, practitioners and technocrats who work in the loosely defined sector of ‘global health.’ The first point relates to the question of whether we should be satisfied with global poverty reduction trends; the second to the threats posed by global warming and ecological degradation.

## The Celebration of Progress

Much of the general satisfaction that is generated around poverty eradication relates to trends in *extreme poverty*. Until 2015, extreme poverty was defined by the World Bank as living on less than $1.25/day. Benatar comments on trends related to an income threshold of $2/day which approximates to the new extreme poverty line set at $1.90/day by the World Bank in 2015.^2^ The shift from $1.25 to $1.90 was made to accommodate changes in the price of basic goods and services and purchasing power.

According to the World Bank, the number of people living in extreme poverty has more than halved since 2001 and is now believed to represent about 10% of the world’s population.^3^ This suggests some progress in poverty reduction, notwithstanding some analysts arguing that the Bank’s methods for standardising ‘purchasing power’ across different countries and currencies under-estimates the true number of people living in extreme poverty.^4^

However, Benatar argues that any reasonable commitment to eradicating poverty should account for all who live in poverty, and not just those in extreme poverty. Although the question of how one defines and measures poverty has exercised economists, political scientists and philosophers for decades, it is hard to imagine anyone disagreeing with the view that anyone living on an income of less than $10/day is poor. And yet, a staggering 71% of the world’s population live below this income line.^5^ By this measure, poverty has been growing across the world, not shrinking. The tendency to focus on the prevalence of *extreme* poverty directs attention away from this fact.

A more important point made by Benatar is that a *celebration* of reductions in extreme poverty has the effect of directing attention away from the more fundamental issue of increasing inequality and widening disparities in wealth and power. This is important because the prevalence of global poverty is largely a consequence of the inequitable distribution of resources and of various forms of structural violence that simultaneously produce wealth and privilege on one hand and poverty and disempowerment on the other.^6^ Fraser argues that the term ‘the global poor’ should be replaced with the term ‘the globally exploited’ or ‘the globally excluded’ so as to explicitly acknowledge the social causes of poverty.^7^ Doing so would leave the global community feeling much less self-satisfied with the limited reductions in the number of people living on less than $2/day.

Some may suggest that Benatar is being overly negative and unappreciative of the fact that levels of extreme poverty have fallen. It may also be pointed out that the global poor have enjoyed other gains, including improvements in health and reductions in mortality rates.^8^ It is also often claimed that the poor have benefited from advancements in democracy and freedom. For example, it is not infrequent for mainstream news journals to celebrate the rising number of parliamentary democracies in Africa,^9^ or to suggest that the internet and new mobile technologies have empowered the global poor,^10^ or that economic globalisation has extended economic freedom and opportunities to all people.^11^ In short, the global poor are not just better off, but also healthier and freer.

Many who work in ‘global health’ tend to share this positive, ‘glass half-full’ picture of human progress. Positive optimism and the celebration of selective indicators of health improvement are distinct features of narratives projected by actors such as the Gates Foundation, the World Bank and the Global Fund. In particular, technological developments in health are lauded as being both cost-effective *and* capable of transforming the lives of the poor.

But Benatar is not suggesting a pessimistic outlook - rather he is calling for a more critical perspective that challenges those narratives that lead away from any discussion of the socially determined maldistribution of wealth (poverty) and health (disease and illness), or which have the effect of concealing the structural violence and injustice that underpins global poverty, even while health indicators are improving for the global poor. There are good reasons for doing so.

As already mentioned, it is arguable that poverty is actually increasing worldwide. Additionally, while there may be a greater number of representative forms of national democracy, the increasingly unequal distribution of wealth and power has created the basis for democratic structures and processes to be corrupted or captured by wealthy elites in many countries. Neoliberal globalisation, including the rise in power of transnational corporations and global finance, and the consequent weakening of national sovereignty (especially in poor countries), have also impinged the ability of the majority poor to enjoy the theoretical benefits of the expansion of democratic elections across the world.

In terms of the reductions in mortality rates, a more critical perspective is warranted if we recognise the fragility of recent global health gains and the threats posed by climate change and ecosystems collapse, anti-microbial resistance, and the prospect of growing levels of violence and armed conflict across the globe. In other words, the predominantly biomedical approach that prevails in global health and which has undoubtedly improved our ability to keep people alive for longer in conditions of poverty, may eventually fail in the medium to long term if we neglect the social determinants of both human health *and* environmental degradation.

Finally, notwithstanding the reductions in mortality, the fact that such large proportions of the world’s population live in social, economic and environmental conditions that are inconsistent with a good life also suggests a need for a more critical approach that places equity at the heart of how we measure progress.

## Nature and the Planet

Benatar also calls for a more urgent recognition of the dangers posed by climate change and ecological degradation, and for humanity to abandon its human-centred model of development in favour of one that places the planet and nature at the centre of our imaginations.

Many of us already know that global warming, climate and weather changes, biodiversity loss and ocean acidification present an existential threat to humanity. High profile Lancet publications of reports from a Commission on Climate Change and Health and a Commission on Planetary Health, together with Margaret Chan asserting that climate change is ‘the defining issue’ of the 21st century,^12^ would suggest that the health community is responding adequately to the problems of excessive greenhouse gas emissions and consumption patterns that are degrading the planet’s capacity to sustain organised human life.

The reality, however, is that many of us still live beyond our fair share of the planet’s capacity and do not yet see unsustainable consumption and lifestyles as a form of ‘ecologically-mediated’ structural violence that is destroying the prospects of future generations and harming the lives of hundreds of millions of mostly poor people who are already experiencing the consequences of climate change. Although we are, to some degree, trapped within a system built around fossil fuel and the idea of perpetual ‘economic growth,’ we also choose to exceed our fair share of the world’s carbon budget by, for example, flying more than we need to, or choosing diets that are patently ecologically unsustainable.

There may be several reasons for this apparent paradox between what we know and what we do. It may be that the scale of danger posed by climate change is under-appreciated, enabled in part by vast amounts of manufactured disinformation that has been generated by the fossil fuel industry and climate denialists. Similarly, it may represent a cognitive-behavioural dissonance that results from an effective and ubiquitous advertising industry that drives a demand for unsustainable material consumption. It may also be that we feel a degree of entitlement from our work to improve the health of the global poor that excuses us from changing our lifestyles. Or we may hope that technological solutions will save us from having to change the way we live. Or we may simply lack hope in the ability of humanity to avoid self-destruction.

It is the grave threat posed by climate change and ecological degradation that points to the need to better understand the paradox between what we know and what we do. Unless we do so, the full potential for the global health community to use its unique mandate and authority to catalyse the wider systemic changes that are required may be left unrealised. Once again, the argument for a more critical approach seems justified.

## What to Do?

Benatar’s article throws up a range of large and complex challenges to which there are no simple solutions. But if the argument is accepted that the global health community needs to adopt a more critical approach, what might this mean in practice? Here I suggest three broad steps that should be taken.

First, the global health community needs to engage more fully with a range of under-represented disciplines and subject areas such as economics, international relations, trade, finance, law, geography and the earth sciences. While some public health scholars have been highlighting the importance of these subject areas to global health, the scholarship and efforts that have been rooted in an understanding of the structural, social and ecological determinants of health must no longer be a minority interest, siloed away from the larger part of the global health community that is focused on the science and practical challenges of individual diseases, their proximal causes and their treatment.

The appeal of ‘pragmatic’ technological and technocratic interventions to save lives and promote incremental improvements in population health is undeniably strong when compared to the messy, unpredictable and conflictual world of politics, economics and climate change. It is understandable that health actors are drawn towards ‘fights’ against disease and illness. But ultimately, a vision of global health that is rooted in both justice and sustainability requires the global health community to develop a broader knowledge base and skills set.

But this by itself is not enough. A second requirement is that we engage politically and confront the politics of global health itself. The latter includes understanding the political dimensions of neoliberal theories and assumptions that have dominated thinking over the past fifty years or so and examining how this shapes health and development policy. Of relevance, for example, is Ron Labonté’s argument in this journal that the SDGs are fundamentally flawed because they assume “that the same economic system, and its still-present neoliberal governing rules, that have created or accelerated our present era of rampaging inequality and environmental peril can somehow be harnessed to engineer the reverse.”^13^

This also includes understanding the way unequal power shapes our global health architecture and policy approaches. The many global health partnerships that have emerged over the past two decades, for example, have worked effectively to reconcile the mission of global health actors (from civil society, academia and the United Nations [UN]) with the interests of powerful private actors. Similarly, the emphases within global health on charity and technology as solutions for the afflictions of the global poor, or more recently on ‘health security,’ need to be assessed politically in terms of transformatively redistributing power and wealth, or affirming social justice as a foundation for health and well-being.

Finally, a more critical global health community would recognise the need to achieve global outcomes through local action. New economic models and re-democratisation, for example, are vital ingredients to the systemic change that is required – but these ingredients will only be provided in sufficient quantity if communities, municipalities and other local groupings are actively engaged in their generation. Adequate systemic change, enabled by policy and legislation, will only occur if shaped and driven by demands from the ground. At the same time, systemic change can be catalysed by smaller-scale changes and developments involving communities at the local level. The large number of health professionals and workers who operate at the local level should be central to these endeavours, and those of us working in global health should look to enable our local health counterparts to create progressive change from the bottom up.

## Ethical issues

Not applicable.

## Competing interests

Author declares that he has no competing interests.

## Author’s contribution

DM is the single author of the paper.
